# Critical Role of H_2_O_2_ Generated by NOX4 during Cellular Response under Glucose Deprivation

**DOI:** 10.1371/journal.pone.0056628

**Published:** 2013-03-21

**Authors:** Satoshi Owada, Yuko Shimoda, Katsuya Tsuchihara, Hiroyasu Esumi

**Affiliations:** 1 Department of Integrated Biosciences, Graduate School of Frontier Sciences, The University of Tokyo, Kashiwa, Japan; 2 Cancer Physiology Project, Research Center for Innovative Oncology, National Cancer Center Hospital East, Kashiwa, Japan; Institute for Virus Research, Laboratory of Infection and Prevention, Japan

## Abstract

Glucose is the most efficient energy source, and various cancer cells depend on glycolysis for energy production. For maintenance of survival and proliferation, glucose sensing and adaptation to poor nutritional circumstances must be well organized in cancer cells. While the glucose sensing machinery has been well studied in yeasts, the molecular mechanism of glucose sensing in mammalian cells remains to be elucidated. We have reported glucose deprivation rapidly induces AKT phosphorylation through PI3K activation. We assumed that regulation of AKT is relevant to glucose sensing and further investigated the underlying mechanisms. In this study, AKT phosphorylation under glucose deprivation was inhibited by galactose and fructose, but induced by 2-deoxyglucose (2-DG). Both 2-DG treatment and glucose deprivation were found to induce AKT phosphorylation in HepG2 cells. These findings suggested that glucose transporter may not be involved in the sensing of glucose and induction of AKT phosphorylation, and that downstream metabolic events may have important roles. A variety of metabolic stresses reportedly induce the production of reactive oxygen species (ROS). In the present study, glucose deprivation was found to induce intracellular hydrogen peroxide (H_2_O_2_) production in HepG2 cells. N-acetylcysteine (NAC), an antioxidant reagent, reduced both the increase in cellular H_2_O_2_ levels and AKT phosphorylation induced by glucose deprivation. These results strongly suggest that the glucose deprivation-induced increase of H_2_O_2_ in the cells mediated the AKT phosphorylation. RNA interference of NOX4, but not of NOX5, completely suppressed the glucose deprivation-induced AKT phosphorylation as well as increase of the intracellular levels of ROS, whereas exogenous H_2_O_2_ could still induce AKT phosphorylation in the NOX4-knockdown cells. In this study, we demonstrated that the ROS generated by NOX4 are involved in the intracellular adaptive responses by recognizing metabolic flux.

## Introduction

The supply of nutrients and oxygen is pivotal for cell survival and function, because of the large energy requirements of cells. This need is especially critical during cell proliferation. Proliferation is a process during which the numbers of cells successively double; therefore, the synthesis of nucleic acids, lipids, proteins and sugars is obligatory for successful proliferation. Glucose serves as a carbon source for the synthesis of nucleic acids, non-essential amino acids, lipids, and sugar. The intermediate metabolites in the glycolytic system are indispensable for non-essential amino acid synthesis, and intermediate metabolites and coenzymes in the pentose-5-phosphate pathway are required for the synthesis of nucleic acids and lipids. In addition, glucose is also needed for energy production in all cells.

Because of the pivotal role of glucose in the maintenance of the cellular functions, survival, and proliferation, elaborate mechanisms for detecting glucose availability in the cellular microenvironment exist in cells. The molecular mechanisms involved in the sensing of extracellular glucose concentrations have been extensively studied in yeasts. Yeasts detect the extracellular glucose concentrations using Snf3/Rtg2 (a glucose transporter homolog that has no capability as a transporter). Extracellular glucose causes this sensor to generate an intracellular signal that induces the expressions of several HXT genes encoding hexose transporters. The glucose signal induces HXT gene expression by influencing the function of the Rgt1 transcriptional repressor. In the absence of glucose, Rgt1 is functional and binds to the promoters of the HXT genes, repressing their functions [1], [2], [3]. In contrast, the biochemical basis of the glucose sensing mechanism in mammalian cells is largely unknown.

Meanwhile, most of human cancer tissues are known to be hypoxic, the hypoxia being caused mainly by a poor and heterogeneous blood supply [4], [5], [6], [7]. Glucose as well as oxygen is supplied to cancer tissues via the blood stream, and we assumed that the glucose supply might be limited in human cancer tissues. In fact, the glucose concentrations in human colon cancer and gastric cancer tissues were found to be significantly lower than those in surrounding non-cancerous tissues [8]. In the cancer cells that exist in such environments, the monitoring of and adaptation to extracellular glucose concentrations are assumed to be important for the survival/proliferation of the tumor cells. We previously reported that AKT phosphorylation is immediately enhanced by the absence of glucose and plays a critical role in cellular survival under such condition in various cell lines [9], [10]. AKT can also be activated in response to a variety of cellular stresses, such as heat shock, ultraviolet light irradiation, ischemia, hypoxia, hyperglycemia, and oxidative stress. AKT is a serine and threonine kinase that mediates cell survival under these aforementioned conditions [11], [12], [13], [14], [15].

In the present study, we attempted to elucidate the molecular and biochemical mechanisms involved in the sensing of mammalian cells of the extracellular glucose concentrations, using AKT phosphorylation as an index of the cellular responses to glucose deprivation. We demonstrate the contribution of the H_2_O_2_ generated by NOX4 in the cellular sensing of and adaptation to poor glucose supply.

## Materials and Methods

### Cell cultures

Human fibroblasts derived from the subserosa of the stomach used for this study were kindly gifted to us by Dr Atsushi Ochiai (Pathology Division, Research Center for Innovative Oncology, National Cancer Center Hospital East). Human pancreatic cancer cells (PANC-1), human hepatocellular carcinoma cells (HepG2) and human fibroblasts derived from subserosa of the stomach were cultured in DMEM (GIBCO) supplemented with 10% fetal bovine serum (Biowest). All the cells were purchased from ATCC. The glucose-deprived condition was created as described previously [16].

### Reagents

2′, 7′- Dichlorodihydrofluorescin diacetate (DCFDA) was purchased from Invitrogen. 3′-O-Acetyl-6′-O-pentafluorobenzenesulfonyl-2′,7′-difluorofluorescein (Bes-H_2_O_2_), galactose and fructose were purchased from Wako Pure Chemical Industries. N-acetyl-L-cysteine (NAC) and 2-deoxy-D-glucose (2-DG) were purchased from Sigma Aldrich. LY294002 and PP2 were purchased from Calbiochem.

### Immunoblot analyses

Cells were homogenized in lysis buffer containing 10% SDS (sodium dodecyl sulfate), 10 mM Tris-HCl (pH 7.5) and 1 mM sodium orthovanadate, as described previously [17], and subjected to SDS-PAGE (SDS polyaclylamide gel electrophoresis). The proteins were transferred to a polyvinylidene fluoride microporous membrane (Millipore). The primary antibodies used were: anti-phospho-AKT Ser-473, anti-phospho-SRC Family Tyr-416, and anti-AKT, all obtained from Cell Signaling Technologies, and anti-actin (sc-1615), and c-SRC antibody (SRC2), obtained from Santa Cruz Biotechnology. The anti-OSSA antibody was a kind gift from Dr. Ryuuichi Sakai, National Cancer Center Research Institute. The following secondary antibodies were purchased from Santa Cruz Biotechnology: goat anti-mouse IgG-HRP, goat anti-rabbit IgG-HRP. The immunoblots were scanned using a CanoScan LiDE60 image scanner (Canon).

### siRNA transfection

OSSA, NOX4, NOX5, and non-targeting siRNA were purchased from Invitrogen. For the siRNA experiments, the cells were transfected separately using a non-targeting siRNA or two separate specific siRNAs using Lipofectamine 2000 (Invitrogen).

### RT-PCR

Total RNAs were prepared from the cells using ISOGEN (Nippon Gene), and reverse transcription was performed using superscript VILO (Invitrogen). PCR for human NOX family genes was carried out using the following primers: forward 5′-CTCAGCGGAATCAATCAGCTGTG-3′ and reverse 5′-AGAGGAACACGACAATCAGCCTTAG-3′ for Nox4; forward 5′-ATCAAGCGGCCCCCTTTTTTTCAC-3′ and reverse 5′-CTCATTGTCACACTCCTCGACAGC-3′ for Nox5.

### Measurement of intracellular ROS levels

The cells were treated under various conditions and then incubated in DMEM or glucose-deprived medium containing 5 µM of DCFDA or 5 µM BES-H_2_O_2_-Ac at 37°C for 30 min. Then, the cells were detached from the plate with trypsin/EDTA, washed with PBS, resuspended in 500 µL of PBS, and placed on ice, protected from light. The intensity of the fluorescence of each cell was immediately measured using a FACS CANTO (Becton Dickinson) equipped with an argon ion laser (488 nm excitation). Each experiment was conducted in triplicate, and 10,000 cells per sample were measured. The histogram was analyzed using the software program BD FACS DIVA (Becton Dickinson).

## Results

### AKT activation by glucose deprivation

**Figure 1:**
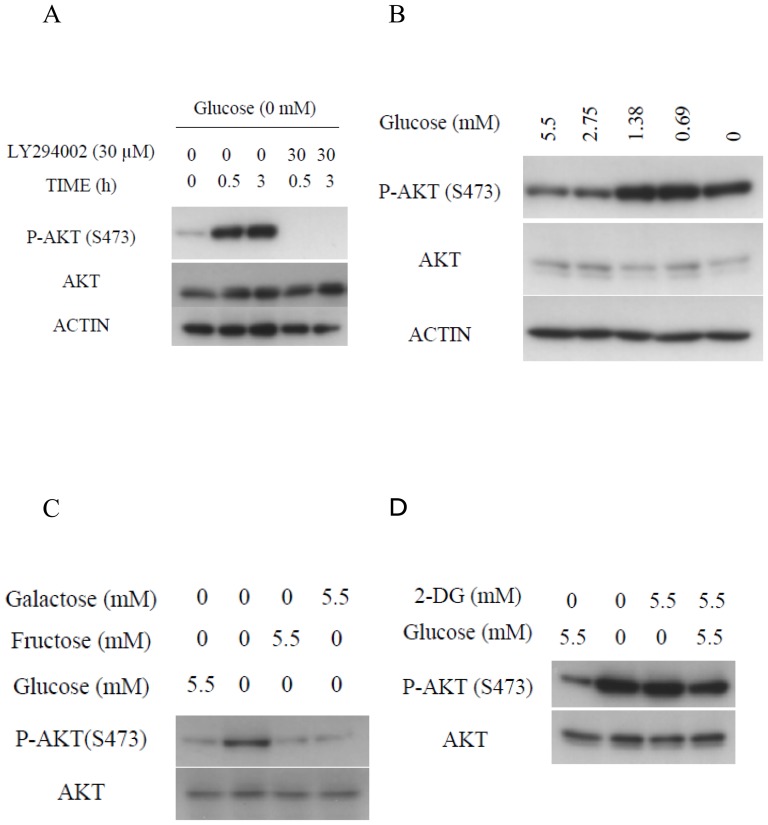
AKT phosphorylation was induced under glucose deprivation. (A) Immunoblotting analyses after incubation of HepG2 cells in the absence or presence of 5.5 mM of glucose and absence or presence of 30 µM of LY294002 for the indicated times. (B) HepG2 cells treated or not treated with various concentrations of glucose for 0.5 h were subjected to immunoblotting. (C) Immunoblotting analyses of HepG2 cells treated or not treated with 5.5 mM of glucose, 5.5 mM of galactose, or 5.5 mM of fructose for 0.5 h. (D) Immunoblotting analyses of HepG2 cells treated or not treated with 5.5 mM of glucose, 5.5 mM of 2-DG, or 5.5 mM of glucose plus 5.5 mM of 2-DG for 0.5 h.

Within 30 minutes, and still after 3 hours, of transferring the HepG2 cells from ordinary DMEM to glucose-deprived medium, AKT was strongly phosphorylated at Ser 473; furthermore, AKT phosphorylation was significantly inhibited by treatment with LY294002 [18], an inhibitor of PI3K (Fig. 1A). Similarly, PI3K-dependent AKT activation was also observed in the pancreatic PANC-1 cells (Fig. S1) in a previous study [10]. Furthermore, increase of AKT phosphorylation induced by glucose deprivation was also observed in human fibroblasts derived from the subserosa of the stomach (Fig. S2).

To examine how glucose deprivation is recognized in these cells, concentration-dependent AKT activation in response to glucose deprivation was examined. When the HepG2 cells were exposed to media containing less than 1.38 mM of glucose, corresponding to one-quarter of the blood glucose level, AKT activation was clearly observed (Fig. 1B). Similarly, an increase in AKT phosphorylation was also observed in PANC-1 cells cultured in the presence of glucose at concentrations of less than 0.69 mM (Fig. S3). To elucidate the glucose sensing mechanism of the cells, the effect of glucose analogues on the AKT activation in response to glucose deprivation was examined. AKT activation was completely inhibited by the addition of either galactose or fructose at a final concentration of 5.5 mM (Fig. 1C). Similar results were observed in the PANC-1 cells (Fig. S4). These observations indicate that AKT is activated by a decrease of some metabolites of glycolysis or metabolic stress, rather than by the decrease of glucose itself. In yeast, the extracellular glucose concentration is sensed by a glucose transporter [1], [2], [3]. To examine whether a similar mechanism may also prevail in mammalian cells, the influence of 2-DG [19], [20] on the AKT phosphorylation induced by glucose deprivation was examined. As shown in Fig. 1D, AKT phosphorylation in the HepG2 cells in response to glucose deprivation was not inhibited by 2-DG. Rather, AKT phosphorylation was clearly induced by the addition of 5.5 mM 2-DG, even in the presence of glucose. This observation indicates that glucose is not sensed by binding to a receptor or transporter, nor is it sensed by hexokinase, because 2-DG can be phosphorylated as efficiently by mammalian hexokinase as glucose. It is possible that the inhibition of binding of some sensors to glucose, if such an interaction occurs, might evoke the same cellular responses as glucose deprivation.

### Role of hydrogen peroxide in the activation of AKT in response to glucose deprivation

**Figure 2:**
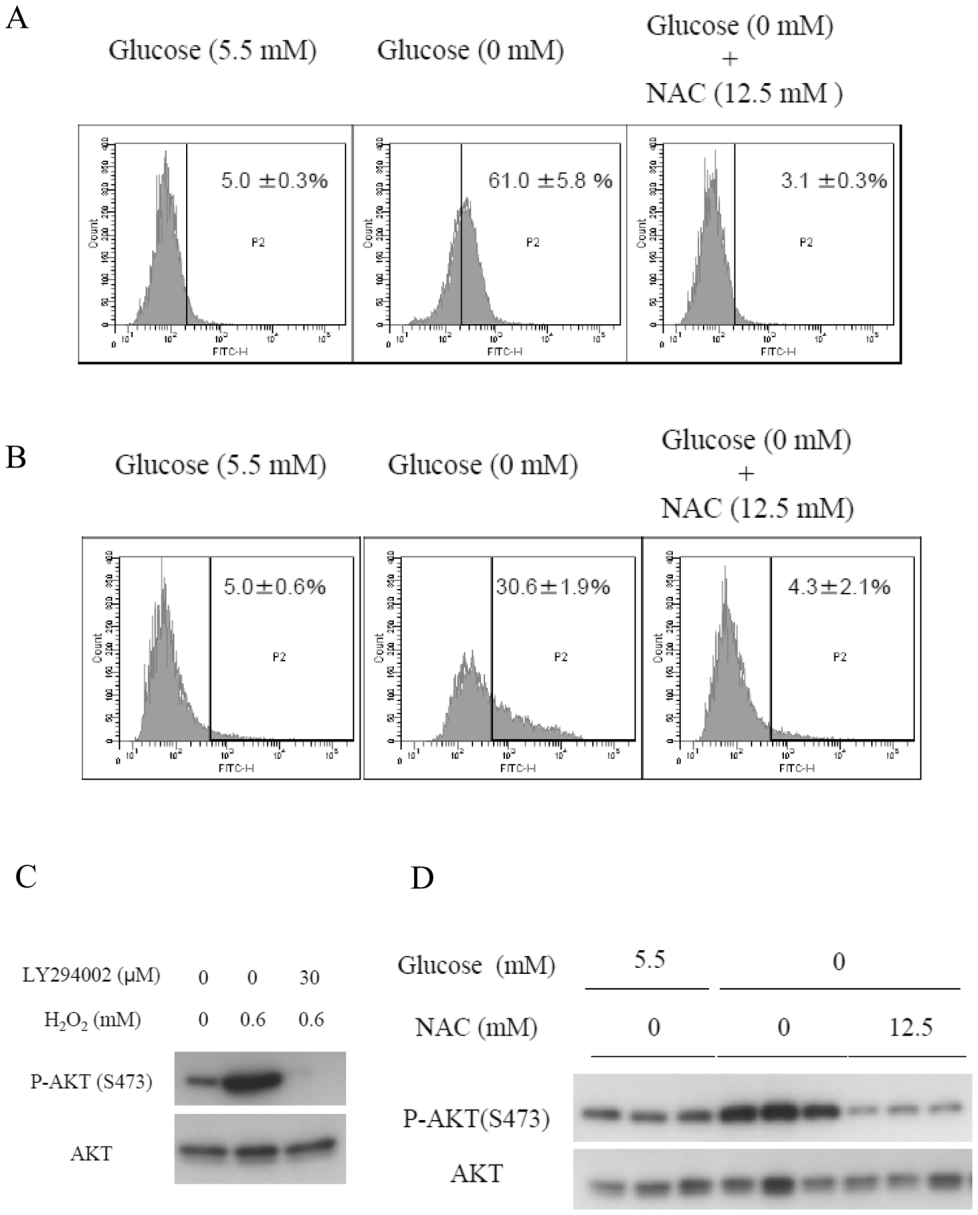
ROS mediates AKT phosphorylation under glucose deprivation. (A)(B)(D) HepG2 cells were cultured in either glucose-containing medium or glucose-deprived medium in the absence or presence of 12.5 mM of NAC for 0.5 h. ROS production was measured using flow cytometry. Cells were stained with (A) 5 µM of DCFDA or (B) 5 µM of BES-H_2_O_2_. Cells were gated within a range contained in the upper 5% of the total cell count under the glucose replete condition. (D) The AKT phosphorylation level was evaluated by immunoblotting. (C) Addition of H_2_O_2_ to media containing 5.5 mM of glucose in the absence or presence of 30 µM of LY294002 for 0.5 h, followed by immunoblotting.

Since AKT phosphorylation in response to glucose deprivation was attenuated by galactose, we assumed that changes in the metabolism might be the cause of the increase in AKT activation. Reactive oxygen species (ROS) are reportedly produced in cells under metabolic stresses [21], [22]. We evaluated the intracellular levels of ROS using dichlorofluorescein diacetate (DCFDA), which measures hydroxyl and peroxyl radicals and other ROS. A significant increase in the intracellular ROS production was observed in the HepG2 cells cultured in glucose-deprived medium treated with DCFDA for 30 minutes (Fig. 2A). 3′-O-acetyl-6′-O-pentafluorobenzenesulfonyl- 2′,7′-difluorofluorescein (BES-H_2_O_2_) specifically detects an increase in the amounts of hydrogen peroxide (H_2_O_2_) [23] in cells treated under the same conditions (Fig. 2B). An increase in the production of ROS induced by glucose deprivation was also observed in the PANC-1 cells and human fibroblasts derived from the subserosa of the stomach (Fig. S5,S6). Addition of galactose or fructose completely prevented the H_2_O_2_ increase (Fig. S7). These results clearly showed that H_2_O_2_ production is induced by glucose deprivation. To elucidate the causal relationship between H_2_O_2_ production and AKT phosphorylation, the effect of addition of exogenous H_2_O_2_ on AKT phosphorylation was examined. Exogenous H_2_O_2_ addition to the culture medium induced PI3K-dependent AKT phosphorylation in a manner similar to glucose deprivation (Fig. 2C). To confirm the causal relation further, the influence of N-acetylcysteine (NAC), an antioxidant reagent, on the AKT phosphorylation induced in the absence of glucose was examined. The addition of NAC to the culture medium at a final concentration of 12.5 mM markedly reduced the ROS levels even under glucose-deprived conditions (Fig. 2A and 2B). Furthermore, the NAC treatment also suppressed the AKT phosphorylation induced by glucose deprivation (Fig. 2D).

### SRC and OSSA are indispensable for AKT phosphorylation induced by glucose deprivation

**Figure 3:**
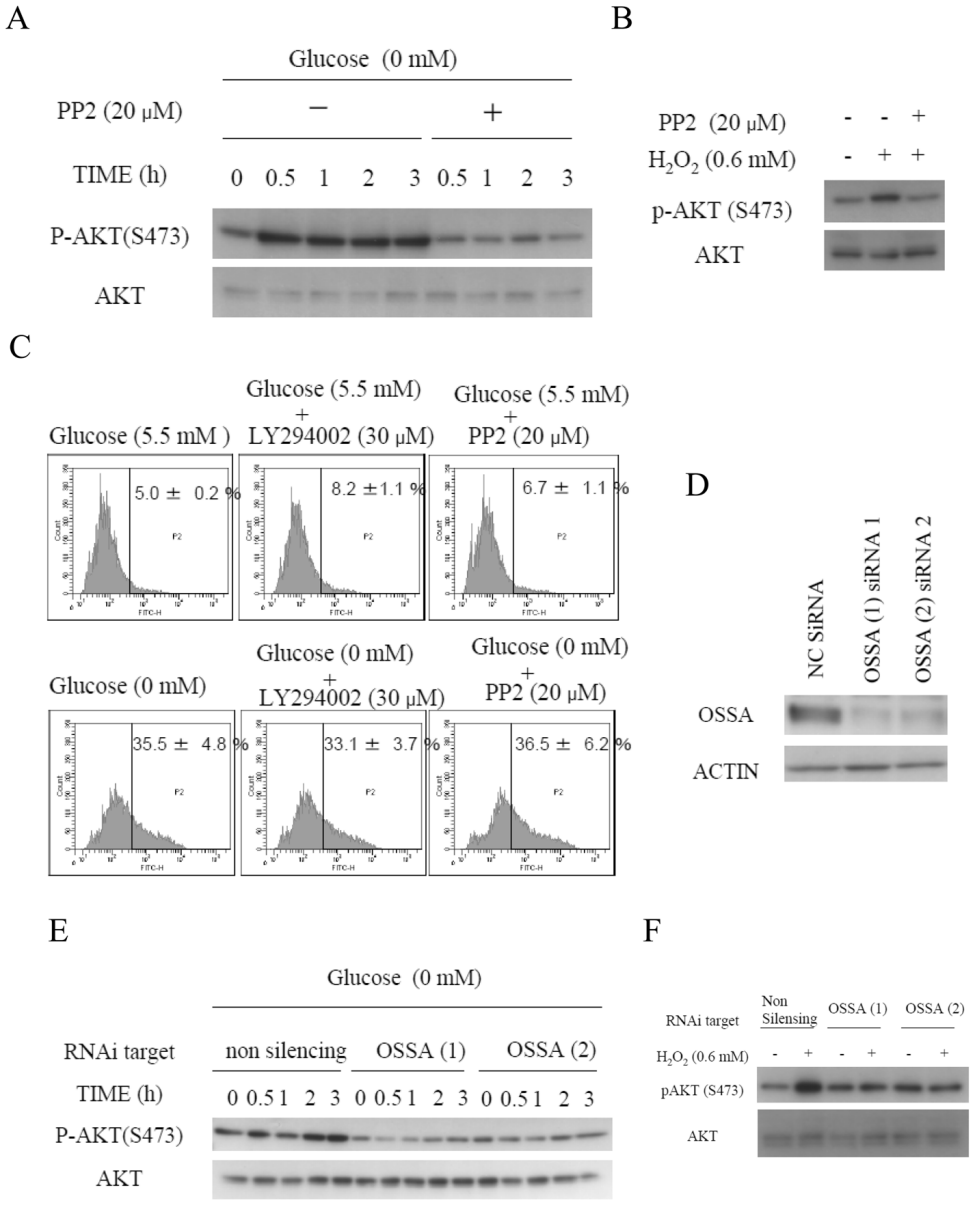
SRC and OSSA are indispensable for the AKT phosphorylation induced by glucose deprivation. (A) Immunoblotting analyses of HepG2 cells in the absence or presence of 5.5 mM of glucose in the and absence or presence of 20 µM of PP2 for the indicated times. (B) Addition of H_2_O_2_ to the culture medium containing 5.5 mM glucosein the absence or presence of 20 µM of PP2 for 0.5 h, followed by immunoblotting. (C) HepG2 cells were cultured in medium containing or not containing (glucose-deprived) 5.5 mM of glucose in the absence or presence of 30 µM of LY204002 or 20 µM of PP2 for 0.5 h. The cells were stained with 5 µM of BES-H_2_O_2_. ROS production was measured using flow cytometry. (D) siRNA-treated HepG2 cells were subjected to immunoblotting analyses using OSSA antibody. (E) Immunoblotting analyses of HepG2 cells transfected with a non-targeting siRNA or two separate OSSA siRNAs in the absence or presence of 5.5 mM of glucose for the indicated times. (F) Addition of H_2_O_2_ to the medium of OSSA-knockdown cells containing 5.5 mM glucose for 0.5 h, followed by immunoblotting.

SRC is involved in an alternate PI3K-activating pathway, and OSSA, a scaffold protein also known as FAM120A, reportedly activates the SRC-PI3K pathway in the presence of oxidative stress [24]. Thus, the involvements of SRC and OSSA in the glucose deprivation-induced phosphorylation of AKT were examined. PP2, a specific SRC family inhibitor [25], clearly inhibited the AKT phosphorylation induced by glucose deprivation (Fig. 3A). PP2 also inhibited AKT phosphorylation induced by exogenous H_2_O_2_ (Fig. 3B). Consistent with these findings, PP2 also suppressed the phosphorylation of SRC induced by glucose deprivation and exogenous H_2_O_2_ (Fig. S8). PP2 treatment did not alter the increased ROS levels in HepG2 cells cultured under glucose-deprived conditions (Fig. 3C). Similarly, LY294002 treatment inhibited AKT phosphorylation, but did not alter the ROS production (Fig. 1A, 3C). Suppression of OSSA expression by RNA interference inhibited the AKT phosphorylation induced by glucose deprivation and exogenous H_2_O_2_ (Fig. 3D, 3E and 3F). Thus, SRC and OSSA were concluded as being mediators of the H_2_O_2_ signals induced by glucose deprivation that activate the PI3K-AKT axis.

### NOX4 knockdown inhibits hydrogen peroxide generation under glucose-deprived conditions

**Figure 4:**
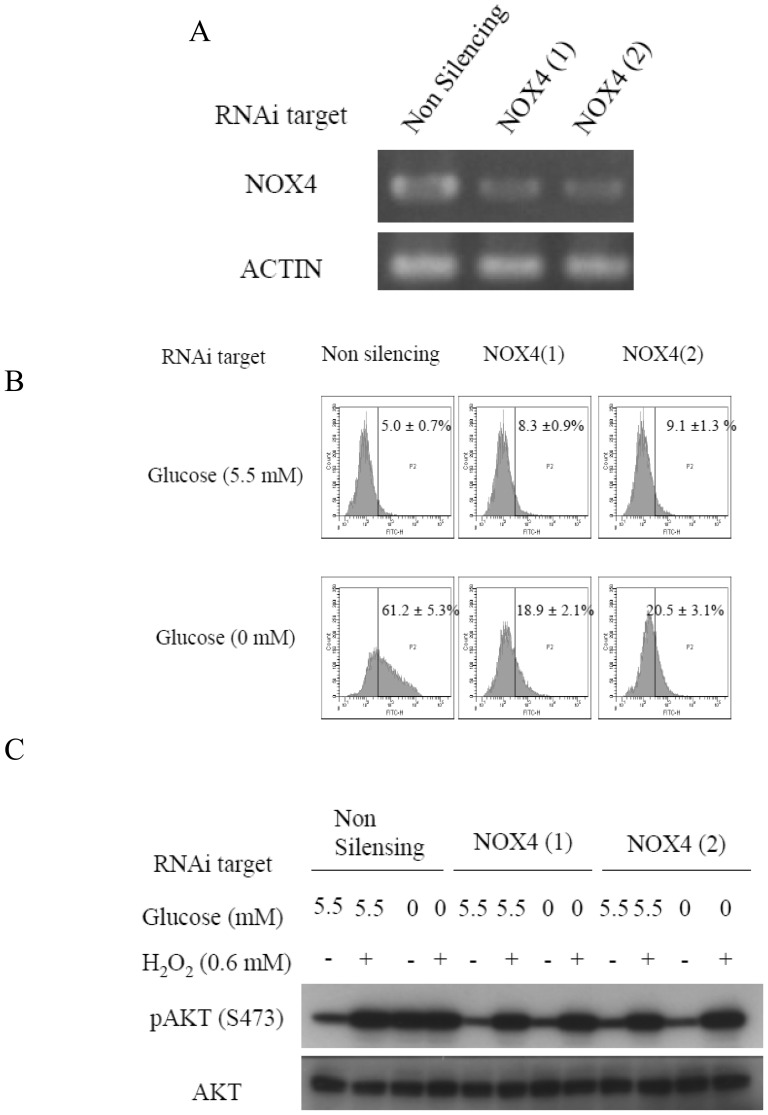
Induction of AKT phosphorylation under glucose deprivation is mediated by ROS generated by NOX4. (A) siRNA-treated HepG2 cells were subjected to reverse-transcriptase PCR (RT-PCR) to confirm NOX4 knockdown. (B) NOX4 knockdown HepG2 Cells were stained with 5 µM of BES-H_2_O_2_ in the absence or presence of 5.5 mM of glucose for 0.5 h. ROS production was measured using flow cytometry. (C) Immunoblotting analyses of HepG2 cells transfected with a non-targeting siRNA or two separate NOX4 siRNAs in the absence or presence of 5.5 mM of glucose or treatment with exogenous H_2_O_2_ for 0.5 h.

NOX4, one of the members of the NADPH oxidase family, is known to be closely involved in the production of ROS in response to growth factor stimuli [26]. Thus, its involvement also in glucose deprivation-induced AKT phosphorylation was examined. RNA interference selectively reduced the expression of NOX4 in HepG2 cells (Fig. 4A). Increase of intracellular ROS levels by glucose deprivation was suppressed by NOX4 knockdown (Fig. 4B). Consistent with this finding, AKT phosphorylation was also not induced in the NOX4 knockdown cells, while exogenous H_2_O_2_ clearly induced AKT phosphorylation in the cells (Fig. 4C). Similar results were obtained in the PANC-1 cells (Fig. S9A, B). PANC-1 cells express NOX5 as well as NOX4, however, knockdown of NOX5 did not alter the AKT phosphorylation level (Fig. S10A, B).

## Discussion

In this study, we tried to elucidate the mechanism of sensing of the extracellular glucose concentration by cells, using AKT phosphorylation as a marker. As reported previously, AKT phosphorylation is induced by glucose deprivation [9], [10]. In addition, increase in AKT phosphorylation has also been confirmed in HepG2 cells cultured in media containing one-quarter of the normal physiological glucose concentration. This fact suggests that cells have sophisticated mechanisms for monitoring extracellular glucose levels. In another study, increase in AKT phosphorylation was confirmed in PANC-1 cells cultured in the presence of glucose levels that are one-eighth of the normal physiological condition. The difference in the minimal trigger concentration of glucose between the HepG2 cells and PANC-1 cells could be related to differences in the origins of the cells or differences in the microenvironments of the tumors the cells were derived from.

In the present study, increase in ROS production was observed by 30 minutes after glucose deprivation, both in cancer cells and human fibroblasts. Thus, it became evident that the mechanism of ROS production under glucose deprivation is preserved in not only cancer cells, but also human fibroblasts. ROS was strongly suspected to mediate the AKT phosphorylation, because AKT phosphorylation was inhibited by treatment with NAC. As H_2_O_2_ has a low selectivity for downstream molecules, it may be involved in the regulation of numerous signaling pathways [27], [28], [29]. Among them, the regulation of AKT phosphorylation, as reported here, is particularly intriguing. AKT mediates cell proliferation and survival [30], [31]. In our previous work, Akt activation was found to play a critical role in cell survival under glucose deprivation [10]. Furthermore, OSSA knockdown and the inhibition of SRC by PP2 suggests that these two elements are fundamental to AKT phosphorylation induced by glucose deprivation. It has been reported that SRC family kinases as their redox sensitive cysteines are the targets of specific oxidation by various oxidants, including H_2_O_2_ [32]. In this study, we clarified that PP2, a specific SRC inhibitor, inhibited AKT phosphorylation induced by glucose deprivation and exogeneous hydrogen peroxide. Thus, SRC is a strong candidate as a hydrogen peroxide sensor. Since PP2 inhibits SRC and other members of the SRC family, we should be careful before denying the relevance of other SRC family kinases [25]. Further investigations, such as by knockdown of individual SRC family kinases will be needed to identify the relevant Src-family kinase.

As with most intracellular signaling cascades, cross-talk and feedback interactions contribute to the overall regulation of PI3K/AKT signaling. S6 kinase-1, a downstream effector of mTORC1, is known to be involved in a negative feedback loop of AKT activation. S6 kinase phosphorylates and inhibits upstream insulin receptor substrate proteins, which diminishes signaling through the PI3K/AKT pathway [33]. We observed that S6 kinase-1 phosphorylation was suppressed in PANC-1 cells under glucose deprivation (unpublished data), suggesting that the negative feedback machinery could be another mechanism regulating AKT phophorylation in cells under glucose deprivation. Furthermore, it was considered that the NADPH/NADP and ATP/AMP ratios may possibly change under glucose deprivation. Therefore, we measured the NADPH/NADP and ATP/AMP ratios; however, no significant changes were observed in at least the first 30 minutes. We also examined the effect of AMPK activation induced by AICAR on AKT activation and the cellular levels of hydrogen peroxide level, but again no significant changes were observed (unpublished data).

AKT phosphorylation in response to glucose deprivation was also completely inhibited following the addition of galactose or fructose instead of glucose. Galactose and fructose enter the glycolytic pathway after they have been metabolized intracellularly to glucose-6-phosphate and fructose-1 or 6-phosphate, respectively. Therefore, the contribution of decrease in metabolites downstream of fructose-1 or 6-phosphate to the induction of AKT phosphorylation under glucose deprivation was hypothesized.

To examine the contribution of the mitochondria, which are the major loci of ROS production, PANC-1 Rho^0^ cells depleted of mitochondrial DNA were produced. When the Rho^0^ cells were exposed to glucose-deprived medium, a large amount of intracellular H_2_O_2_ was produced. As pyruvic acid alone did not inhibit the ROS production completely, we could not assess the contribution of the mitochondria to the induction of ROS production by glucose deprivation further by this method (Shimoda et al. unpublished data). We then studied the involvement of NOX4 as another major locus of ROS production. AKT phosphorylation induced by glucose deprivation was not observed after NOX4 knockdown; no increase in the intracellular ROS levels was observed either, indicating the involvement of NOX4 in the intracellular accumulation of ROS. The contribution of NOX4, but not NOX5, in the signaling triggered by glucose deprivation was rather unexpected. Interestingly, a previous study reported that NOX4 regulates the survival of PANC-1 cells via ROS/ASK1/AKT signaling [34]. It might also be involved in cell survival under glucose-deprived conditions. With respect to the regulation of their activities, there are fundamental differences among the NOX isoforms. Most NOX family members are reportedly switched on and off by their regulatory subunits. NOX4 also functions as a complex with p22phox on internal membranes to produce ROS [35], [36]. NOX4, unlike other members of the NOX family, is known to constitutively induce the production of large amounts of H_2_O_2_, however, the possibility of growth factor signaling being mediated by NOX4 has also been suggested [37]. The results of the present study also suggested that the activity of NOX4 might be regulated. In the present study, glucose deprivation increased the cellular levels of H_2_O_2_, which was suppressed by frucotose and galactose, indicating that NOX4 might be activated by deprivation of some glycolytic intermediate or some downstream products, such as of the pentose phosphate shunt and/or TCA cycle. The results obtained with the use of 2-DG are consistent with this idea. Whether the ROS accumulation under glucose deprivation is caused by increased production of ROS as a result of enhanced activity of NOX4, or by decreased antioxidant capacity, such as that associated with deficient activities of catalase, glutathione peroxidase, and glutathione needs to be further investigated. The intracellular amount of ROS is determined by the activity of the enzymes and the amounts of the substrates available. Therefore, metabolomic analysis of the entire set of metabolites is desired.

In the present study, we found that cells sense and respond to metabolic flux rather than glucose itself, and NOX4 and its product, ROS, play important roles in the cellular adaptive responses.

## Supporting Information

Figure S1 **Immunoblotting analyses after incubating PANC-1 cells in the absence or presence of 5.5 mM of glucose in the absence or presence of 30 µM of LY294002 for the indicated times.**
(TIF)

Figure S2 **Immunoblotting analyses after incubating human fibroblasts derived from subserossa of stomach in the absence or presence of 5.5 mM of glucose for 0.5 h.**
(TIF)

Figure S3 **PANC-1 cells were treated with or without various concentrations of glucose for 0.5 h.**
(TIF)

Figure S4 **Immunoblotting analyses after incubating PANC-1 cells in the absence or presence of 5.5 mM of glucose, 5.5 mM of galactose, or 5.5 mM of fructose for 0.5 h.**
(TIF)

Figure S5 **PANC-1 cells were cultured in either glucose-containing medium or glucose-deprived medium for 0.5 h.** Cells were stained with 5 µM BES-H_2_O_2_. ROS production was measured using flow cytometry.
(TIF)

Figure S6 **Human fibroblasts derived from subserossa of stomach were cultured in either glucose-containing medium or glucose-deprived medium for 0.5 h.** Cells were stained with 5 µM BES-H_2_O_2_. ROS production was measured using flow cytometry.
(TIF)

Figure S7 **HepG2 cells were cultured in the absence or presence of 5.5 mM of glucose, 5.5 mM of galactose, or 5.5 mM of fructose for 0.5 h. ROS production was measured using flowcytometry.** Cells were stained with 5 µM of BES-H_2_O_2_.
(TIF)

Figure S8 **Immunoblotting analyses of HepG2 cells in the absence or presence of 5.5 mM of glucose or treatment with exogenous H_2_O_2_ for 0.5 h.**
(TIF)

Figure S9 (A) siRNA-treated PANC-1 cells were subjected to reverse transcriptional PCR (RT-PCR) to confirm NOX4 knockdown. (B) Immunoblotting analyses after incubating PANC-1 cells transfected with a non-targeting siRNA or two separate NOX4 siRNA in the absence or presence of 5.5 mM of glucose for 0.5 h.
(TIF)

Figure S10 (A) siRNA-treated PANC-1 cells were subjected to reverse transcriptional PCR (RT-PCR) to confirm NOX5 knockdown. (B) Immunoblotting analyses after incubating PANC-1 cells transfected with a non-targeting siRNA or two separate NOX5 siRNA in the absence or presence of 5.5 mM of glucose for 0.5 h.
(TIF)
