# Supplementary figures and images for: Critical Role of H2O2 Generated by NOX4 during Cellular Response under Glucose Deprivation
Source: PLoS One. 2013 Mar 21;8(3):e56628. doi: 10.1371/journal.pone.0056628 (PMC3605446; doi:10.1371/journal.pone.0056628)

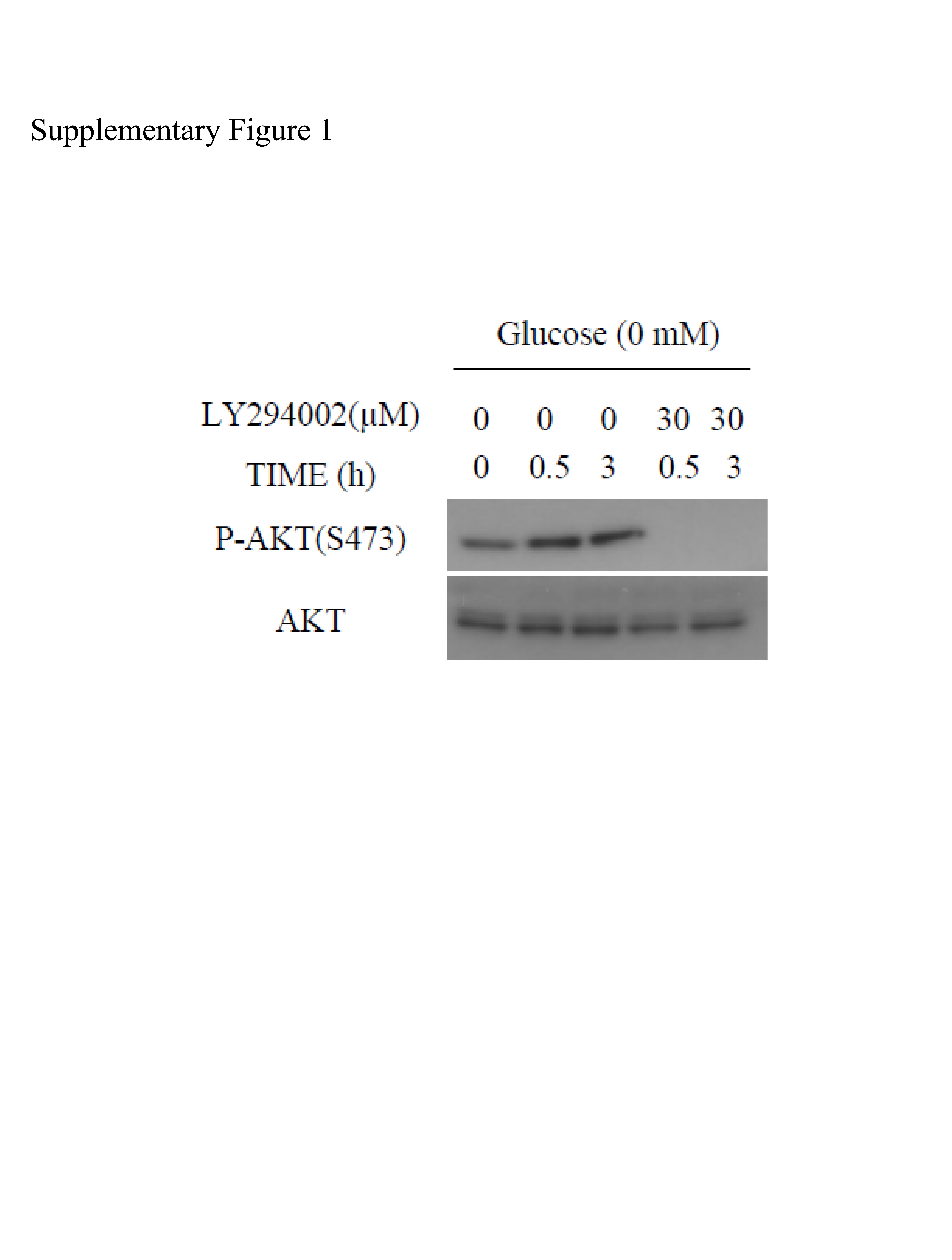

Supplement: Figure S1 — Immunoblotting analyses after incubating PANC-1 cells in the absence or presence of 5.5 mM of glucose in the absence or presence of 30 µM of LY294002 for the indicated times. (TIF) [file pone.0056628.s001.tif]

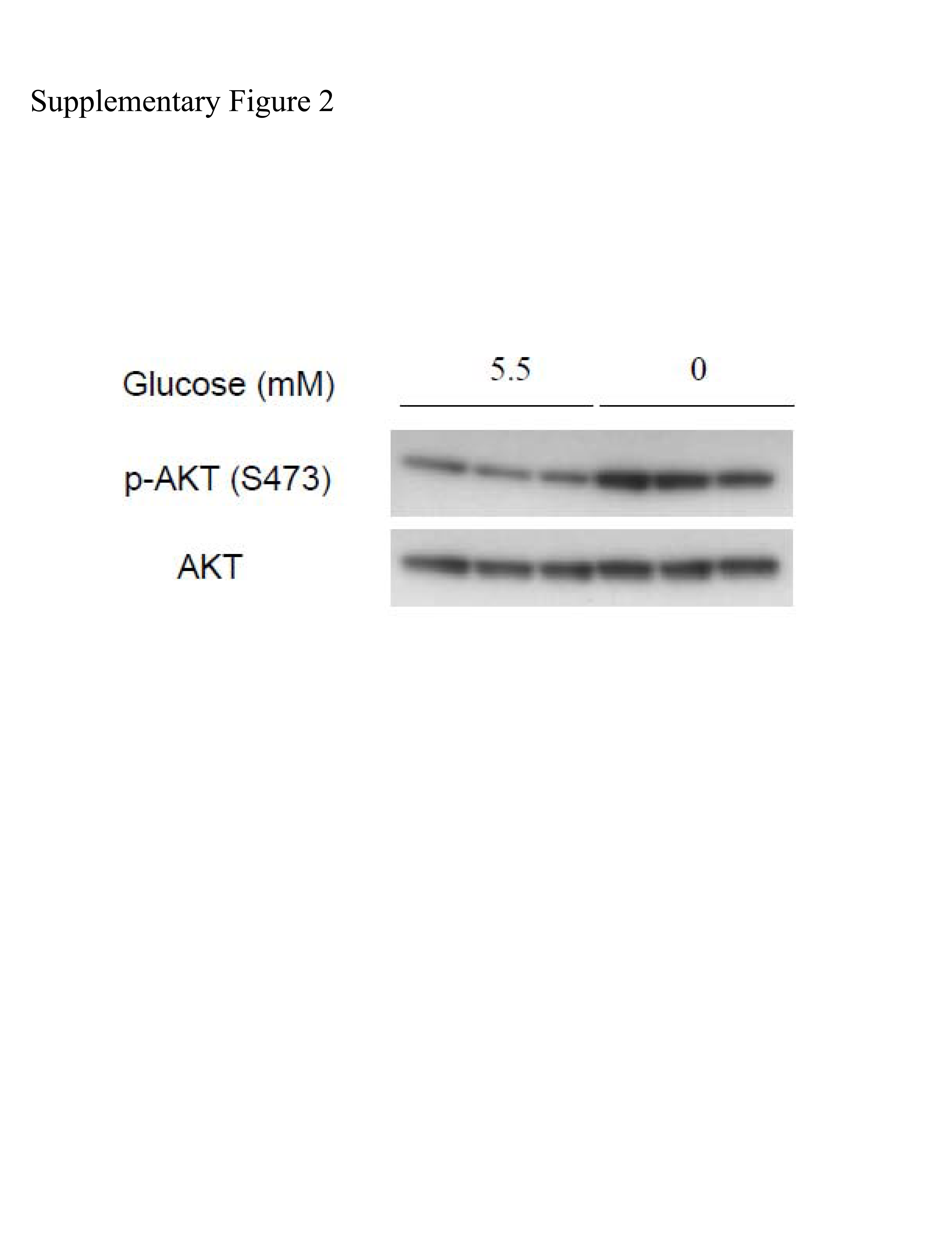

Supplement: Figure S2 — Immunoblotting analyses after incubating human fibroblasts derived from subserossa of stomach in the absence or presence of 5.5 mM of glucose for 0.5 h. (TIF) [file pone.0056628.s002.tif]

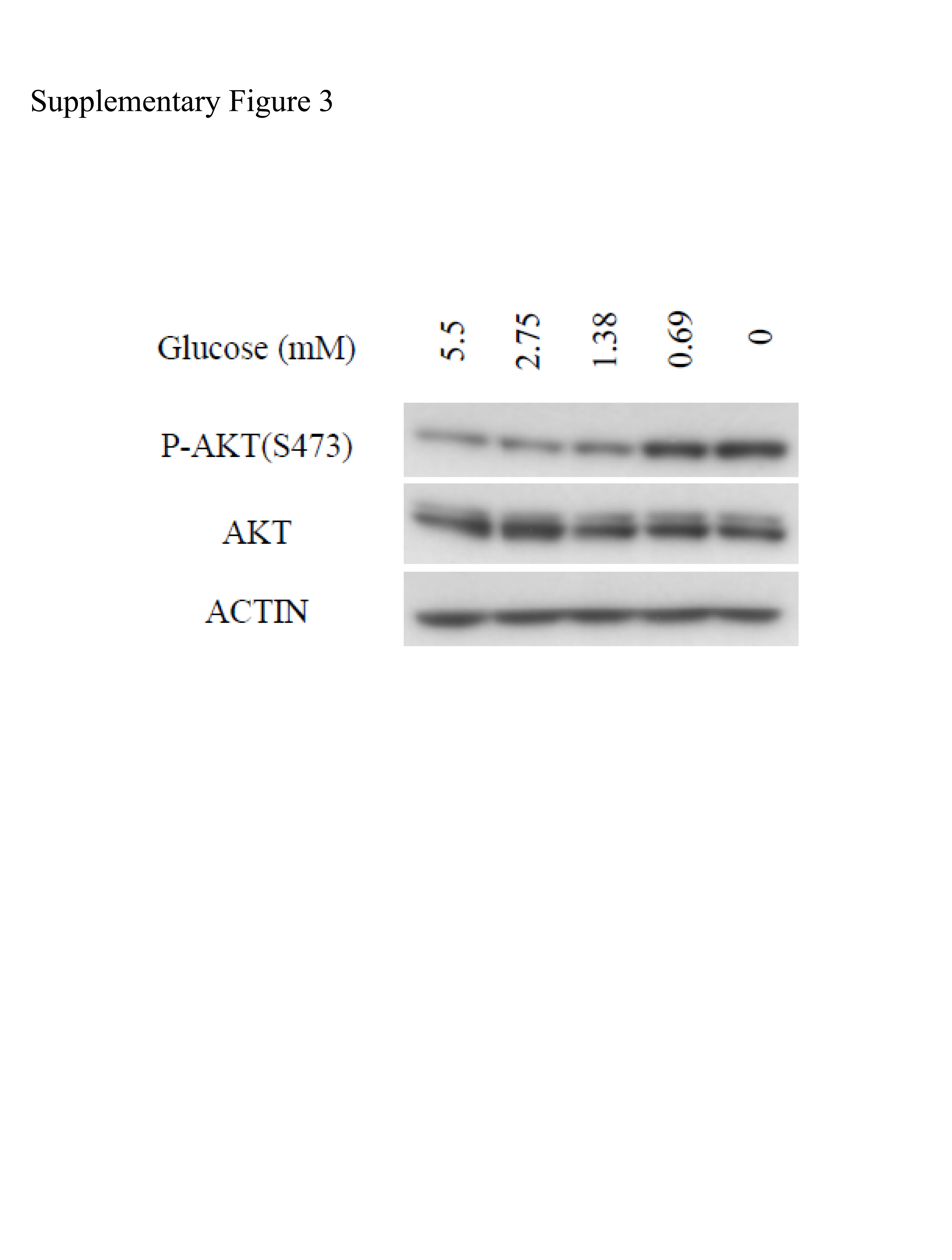

Supplement: Figure S3 — PANC-1 cells were treated with or without various concentrations of glucose for 0.5 h. (TIF) [file pone.0056628.s003.tif]

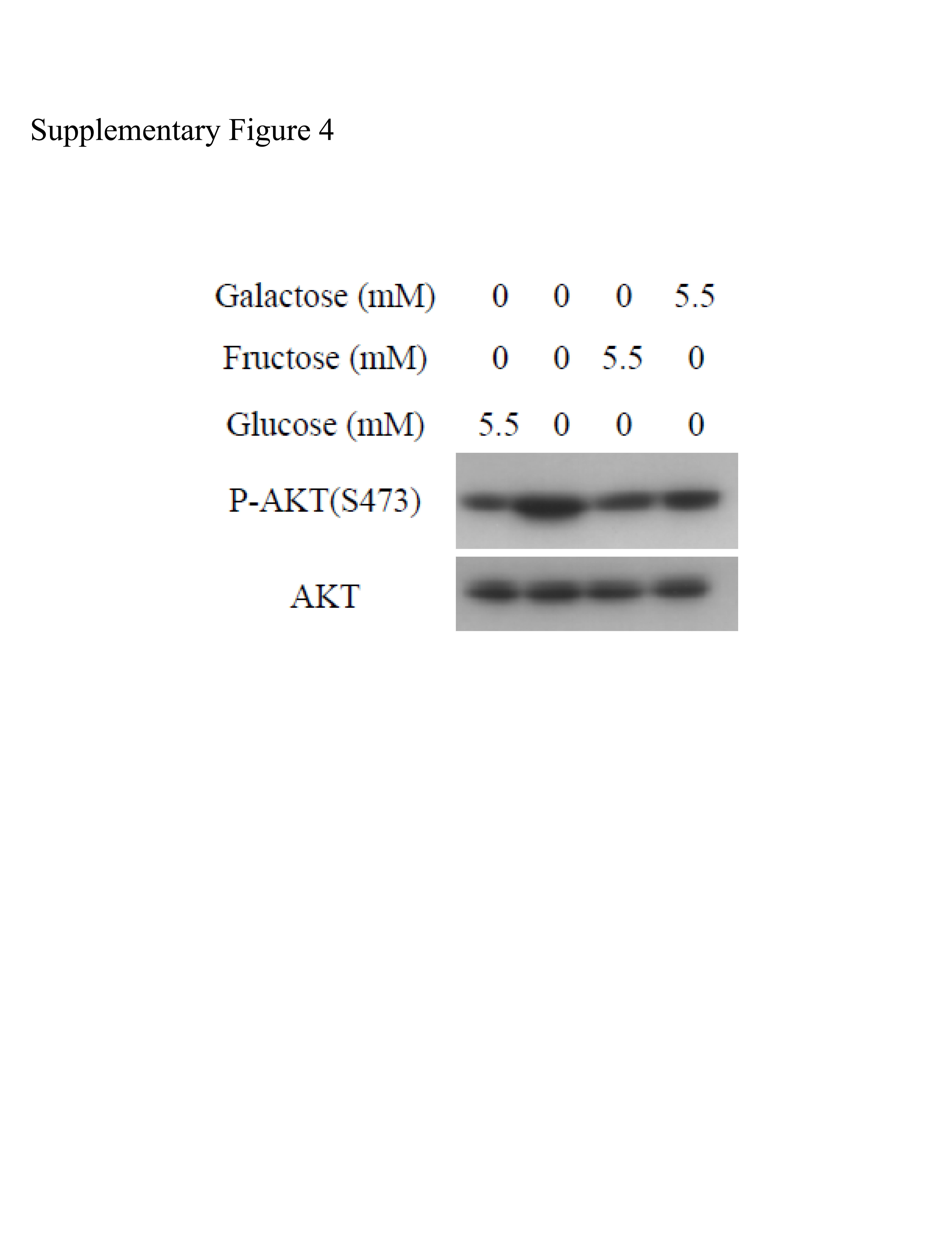

Supplement: Figure S4 — Immunoblotting analyses after incubating PANC-1 cells in the absence or presence of 5.5 mM of glucose, 5.5 mM of galactose, or 5.5 mM of fructose for 0.5 h. (TIF) [file pone.0056628.s004.tif]

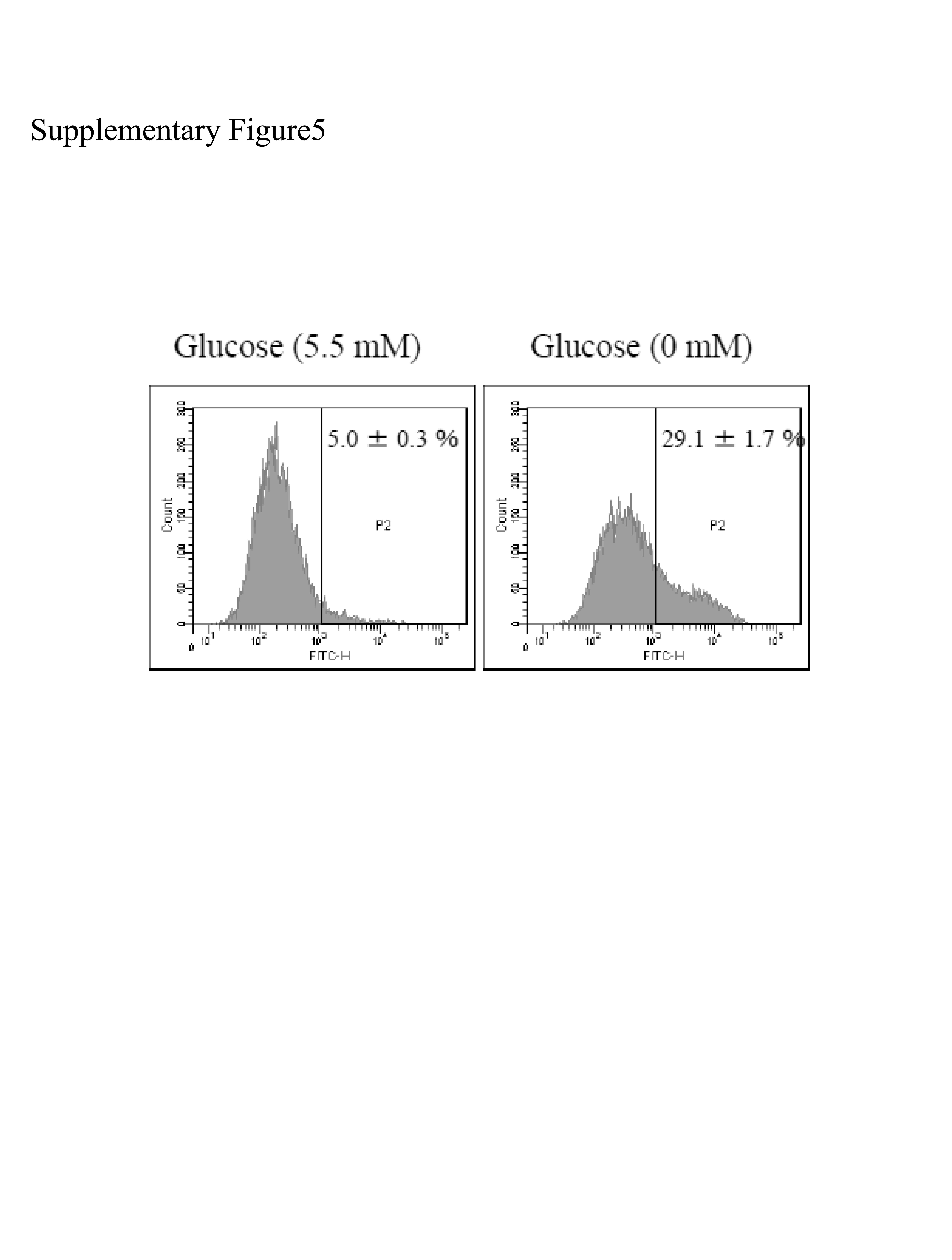

Supplement: Figure S5 — PANC-1 cells were cultured in either glucose-containing medium or glucose-deprived medium for 0.5 h. Cells were stained with 5 µM BES-H2O2. ROS production was measured using flow cytometry. (TIF) [file pone.0056628.s005.tif]

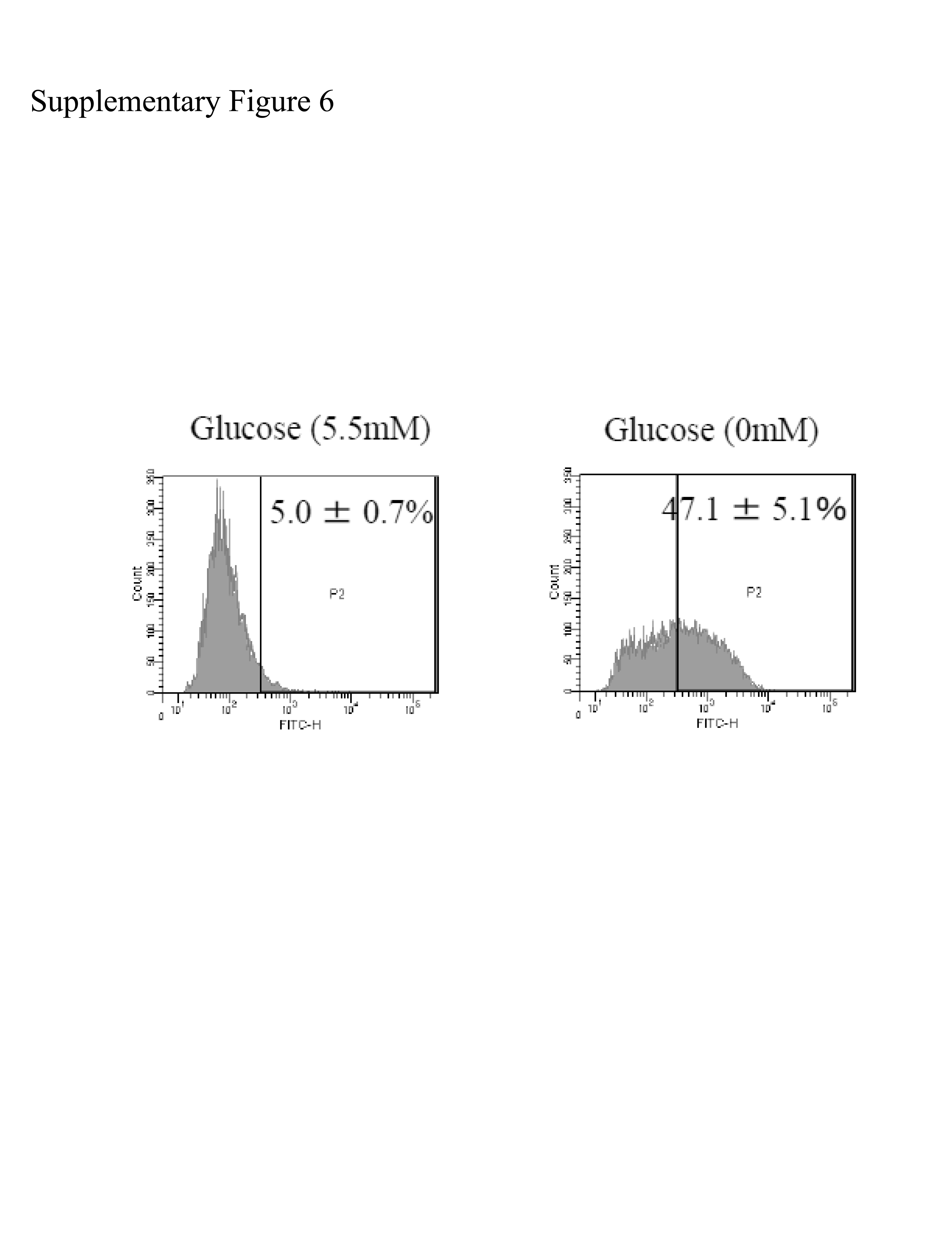

Supplement: Figure S6 — Human fibroblasts derived from subserossa of stomach were cultured in either glucose-containing medium or glucose-deprived medium for 0.5 h. Cells were stained with 5 µM BES-H2O2. ROS production was measured using flow cytometry. (TIF) [file pone.0056628.s006.tif]

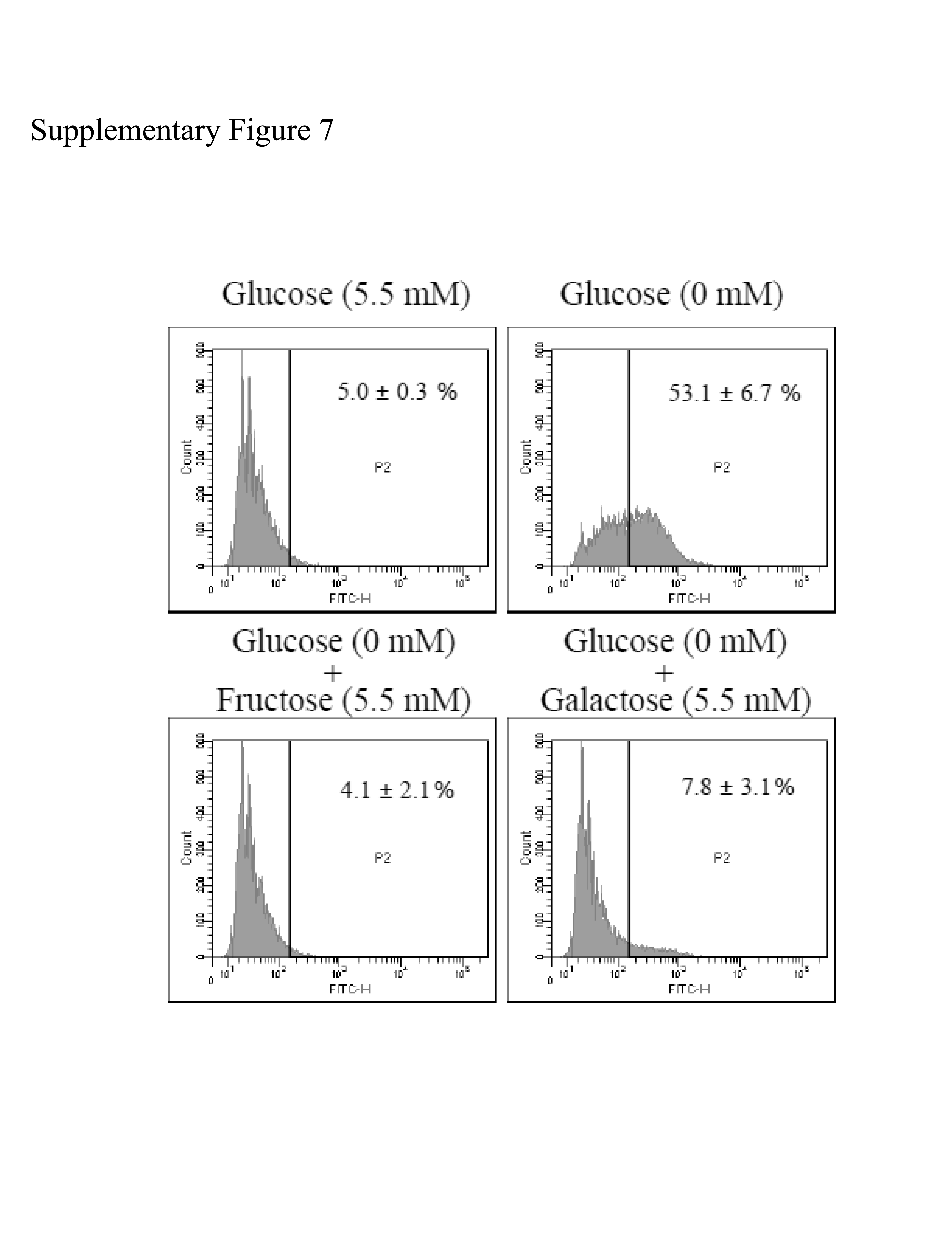

Supplement: Figure S7 — HepG2 cells were cultured in the absence or presence of 5.5 mM of glucose, 5.5 mM of galactose, or 5.5 mM of fructose for 0.5 h. ROS production was measured using flowcytometry. Cells were stained with 5 µM of BES-H2O2. (TIF) [file pone.0056628.s007.tif]

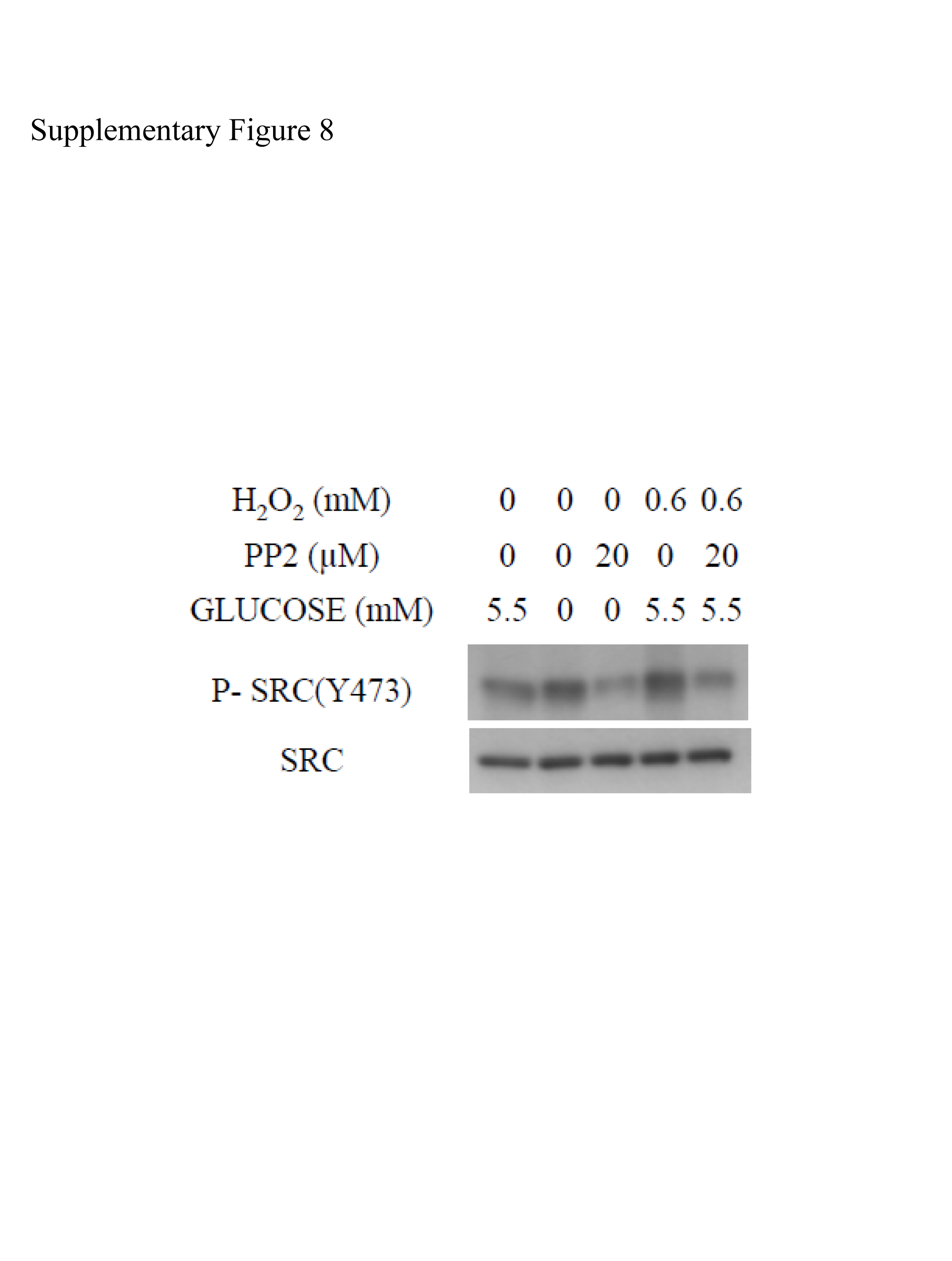

Supplement: Figure S8 — Immunoblotting analyses of HepG2 cells in the absence or presence of 5.5 mM of glucose or treatment with exogenous H2O2 for 0.5 h. (TIF) [file pone.0056628.s008.tif]

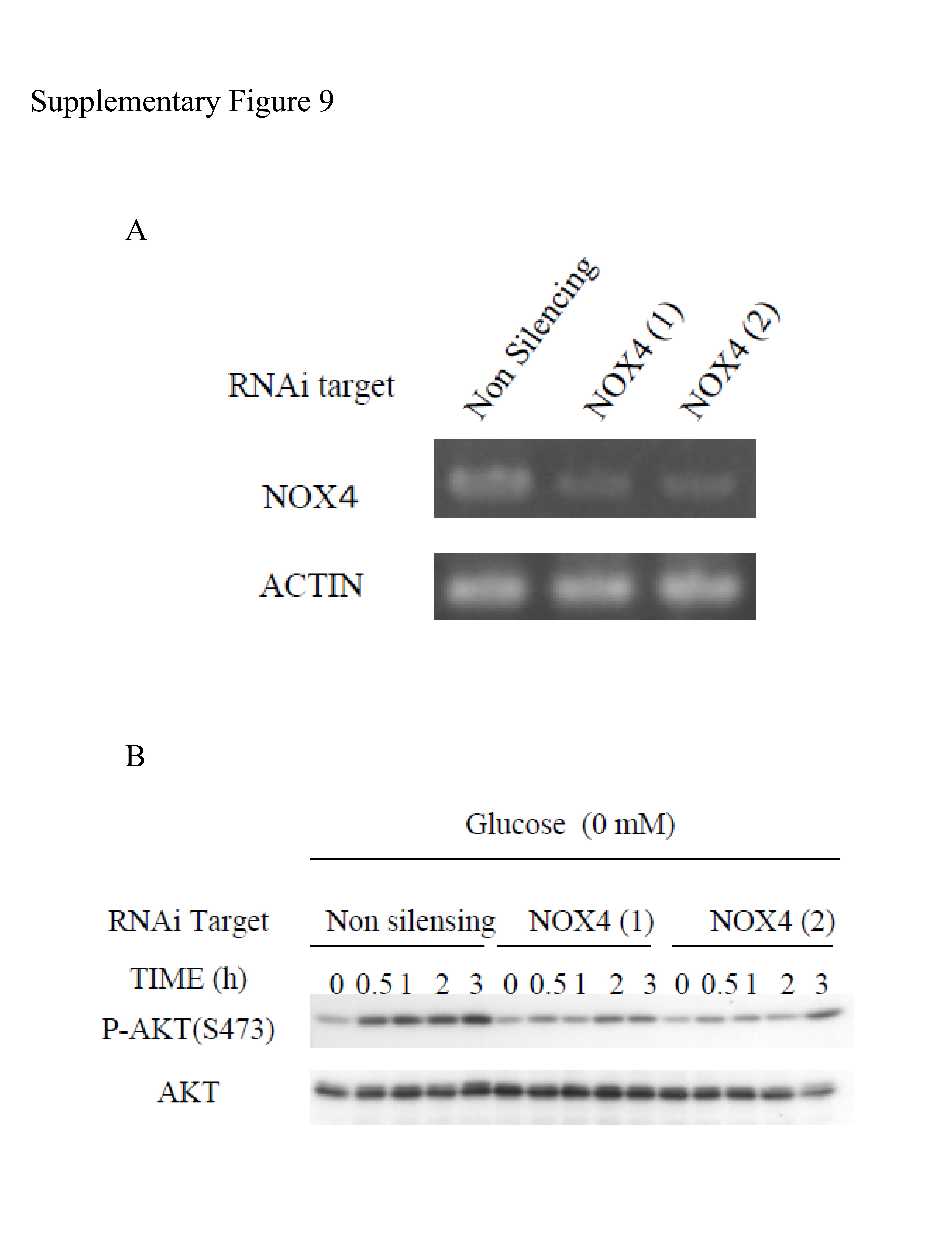

Supplement: Figure S9 — (A) siRNA-treated PANC-1 cells were subjected to reverse transcriptional PCR (RT-PCR) to confirm NOX4 knockdown. (B) Immunoblotting analyses after incubating PANC-1 cells transfected with a non-targeting siRNA or two separate NOX4 siRNA in the absence or presence of 5.5 mM of glucose for 0.5 h. (TIF) [file pone.0056628.s009.tif]

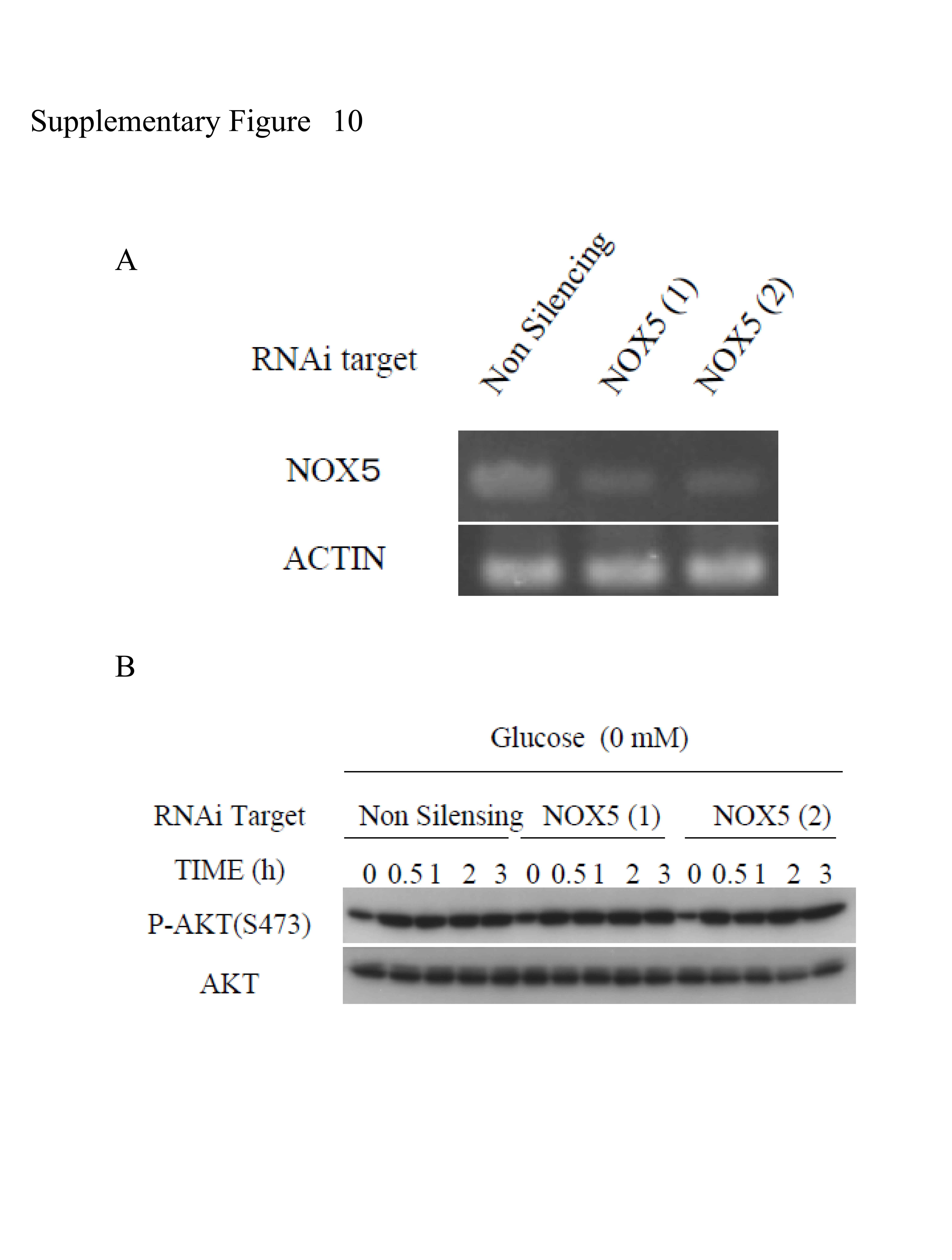

Supplement: Figure S10 — (A) siRNA-treated PANC-1 cells were subjected to reverse transcriptional PCR (RT-PCR) to confirm NOX5 knockdown. (B) Immunoblotting analyses after incubating PANC-1 cells transfected with a non-targeting siRNA or two separate NOX5 siRNA in the absence or presence of 5.5 mM of glucose for 0.5 h. (TIF) [file pone.0056628.s010.tif]
